# Oral HPV-Related Epithelial Proliferation during Fingolimod Therapy: First Case Report and Review of the Literature

**DOI:** 10.1007/s12105-025-01881-0

**Published:** 2026-01-08

**Authors:** Kittiphoj Tikkhanarak, Jay Saepoo, Nidhi Handoo, Emily Lanzel, Sherry Timmons, Ryan Frost, John Hellstein

**Affiliations:** 1 Department of Oral Pathology, Radiology and Medicine, College of Dentistry, The University of Iowa, Iowa City, IA USA; 2 Greenfield Dental, Greenfield, IA USA

**Keywords:** Multiple sclerosis, Fingolimod, Gilenya, HPV, Human papilloma virus, Oral

## Abstract

Fingolimod, an immunomodulatory agent used in the treatment of multiple sclerosis, has been associated with an increased risk of human papillomavirus (HPV)-related mucocutaneous lesions due to its immunosuppressive effects. We report the first documented case of oral mucosal HPV-related epithelial proliferation in a patient undergoing long-term fingolimod therapy. A 36-year-old male presented with multiple mildly papillomatous gingival lesions, during ongoing fingolimod (Gilenya^®^) therapy for eight years. Histopathologic examination revealed parakeratinized stratified squamous epithelium with acanthosis, papillary projections supported by fibrovascular cores, and koilocytosis. HPV DNA testing demonstrated the presence of low-risk HPV types 6/11. The clinical presentation, histopathologic features, and supporting laboratory findings, in the context of long-term immunomodulatory therapy, were consistent with an HPV-related epithelial proliferation potentially associated with fingolimod use. This case expands the anatomic spectrum of HPV-associated epithelial proliferations as a potential adverse effect of fingolimod therapy in patients with multiple sclerosis, broadening clinical awareness beyond the reported cutaneous and anogenital presentations.

## Introduction

Multiple sclerosis (MS) is a chronic autoimmune demyelinating disorder of the central nervous system. Disease-modifying therapies (DMTs) are widely used in relapsing-remitting MS to reduce relapse frequency and delay disability progression. Fingolimod, an oral sphingosine-1-phosphate (S1P) receptor modulator, is one such DMT approved for the treatment of relapsing forms of MS. It functions by sequestering naïve and central memory T cells in lymph nodes, thereby reducing peripheral lymphocyte circulation and exerting systemic immunosuppressive effects [1].

While fingolimod effectively controls MS activity [1], it has been associated with an increased risk of opportunistic infections and virus-related complications, including herpes virus infections, cryptococcosis, histoplasmosis, progressive multifocal leukoencephalopathy, hepatitis C reactivation, and Kaposi sarcoma [2]. Moreover, fingolimod has been implicated in the development of human papillomavirus (HPV)-associated lesions [3–7]. Although such lesions have been documented in the cutaneous and anogenital regions of patients undergoing fingolimod therapy, no prior cases have described involvement of the oral mucosa. Herein, we present the first reported case of oral mucosal HPV-related epithelial proliferation in a patient undergoing long-term fingolimod (Gilenya^®^) therapy.

### Case presentation

**Fig. 1:**
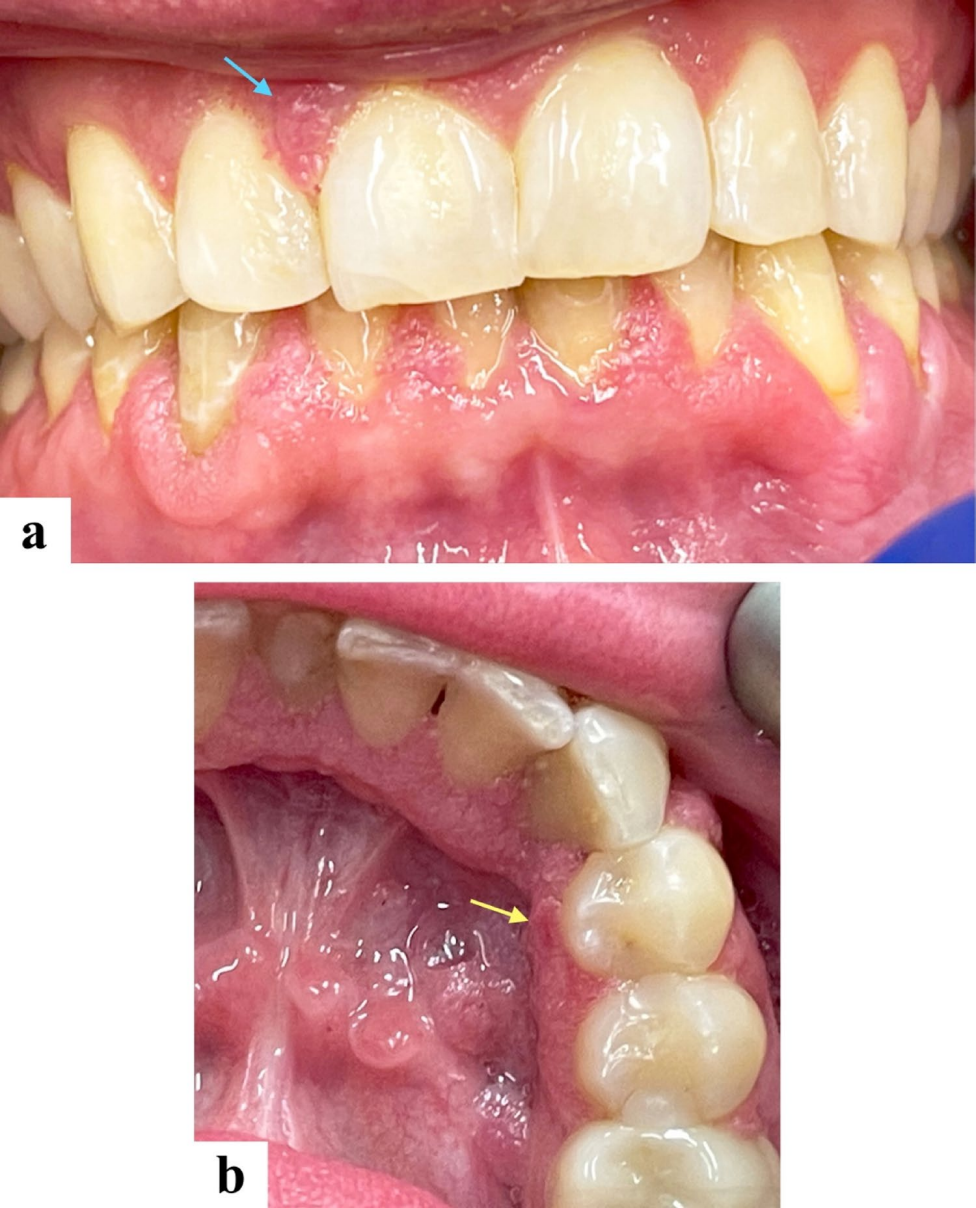
Clinical presentation. Multiple mucosal-colored sessile and pedunculated lesions with a mildly papillomatous surface distributed along **(a)** the labial gingiva of both maxilla (blue arrow) and mandible, and **(b)** the lingual aspect of the anterior and posterior right mandibular gingiva. The yellow arrow indicates the biopsy site

A 36-year-old male presented with numerous painless gingival lesions. He had a known history of multiple sclerosis and had been receiving fingolimod (Gilenya^®^, Novartis Pharmaceuticals Corp., East Hanover, NJ, USA) therapy for eight years. On intraoral examination, multiple sessile and pedunculated mucosal-colored lesions with a mildly papillomatous surface were noted along the labial aspects of both maxillary and mandibular gingiva (Fig. 1a), as well as the lingual anterior and posterior right mandibular gingiva (Fig. 1b).

The clinical differential diagnosis included HPV-related papillomatosis, nevus unius lateris, acanthosis nigricans, and focal dermal hypoplasia syndrome. Given the patient’s history of long-term immunosuppressive therapy and absence of cutaneous, systemic, or syndromic findings, HPV-related papillomatosis associated with fingolimoid therapy was considered the most plausible clinical diagnosis.

**Fig. 2:**
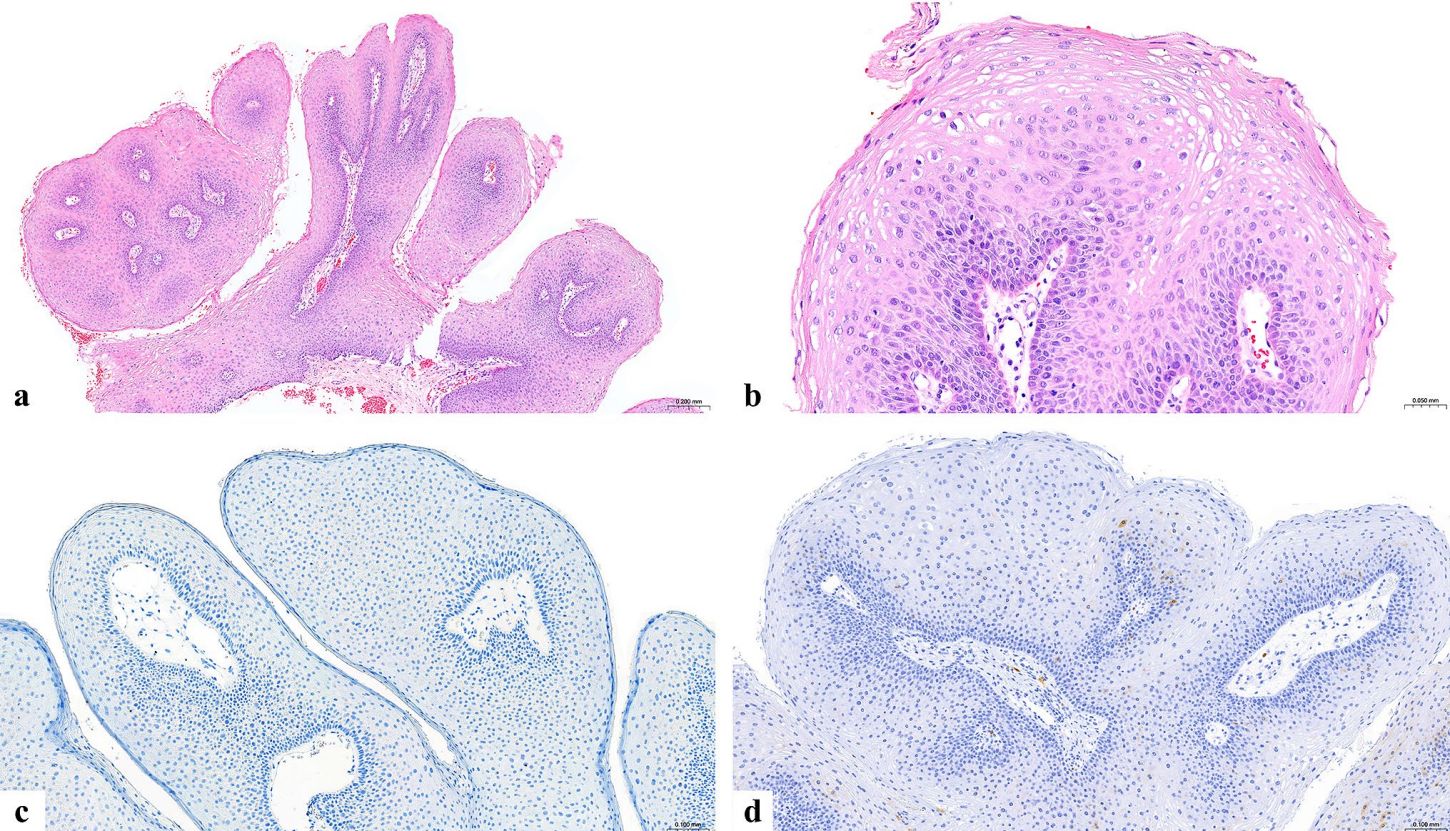
Histopathologic features of the current lesion. **(a)** Exophytic acanthotic, parakeratinized stratified squamous epithelial proliferation with finger-like projections supported by thin fibrovascular cores (hematoxylin and eosin [H&E], ×50). **(b)** Koilocytosis in the superficial spinous layer (H&E, ×200). **(c)** In situ hybridization for HPV16 (×100) and **(d)** p16 immunohistochemistry (×100), both demonstrating negative

An incisional biopsy was obtained from the lesion lingual to right mandibular first premolar tooth (Fig. 1b). Histopathologic evaluation revealed parakeratinized stratified squamous epithelium with exophytic and acanthotic epithelial proliferation forming finger-like projections supported by thin fibrovascular cores (Fig. 2a). Koilocytosis was observed in the superficial spinous layer (Fig. 2b). In situ hybridization for high-risk HPV type 16 (Fig. 2c) and p16 immunohistochemistry (Fig. 2d) were both negative. Additional PCR-based HPV DNA genotyping was performed on the formalin-fixed paraffin-embedded (FFPE) tissue and demonstrated the presence of low-risk HPV types 6/11, with no detection of high-risk types 16, 18, 31, 33, 45, or 58.

Based on the histopathologic features, additional HPV testing results, and clinical context of long-term immunosuppressive therapy with fingolimod, a diagnosis of HPV-related epithelial proliferation associated with fingolimod therapy was favored.

**Fig. 3:**
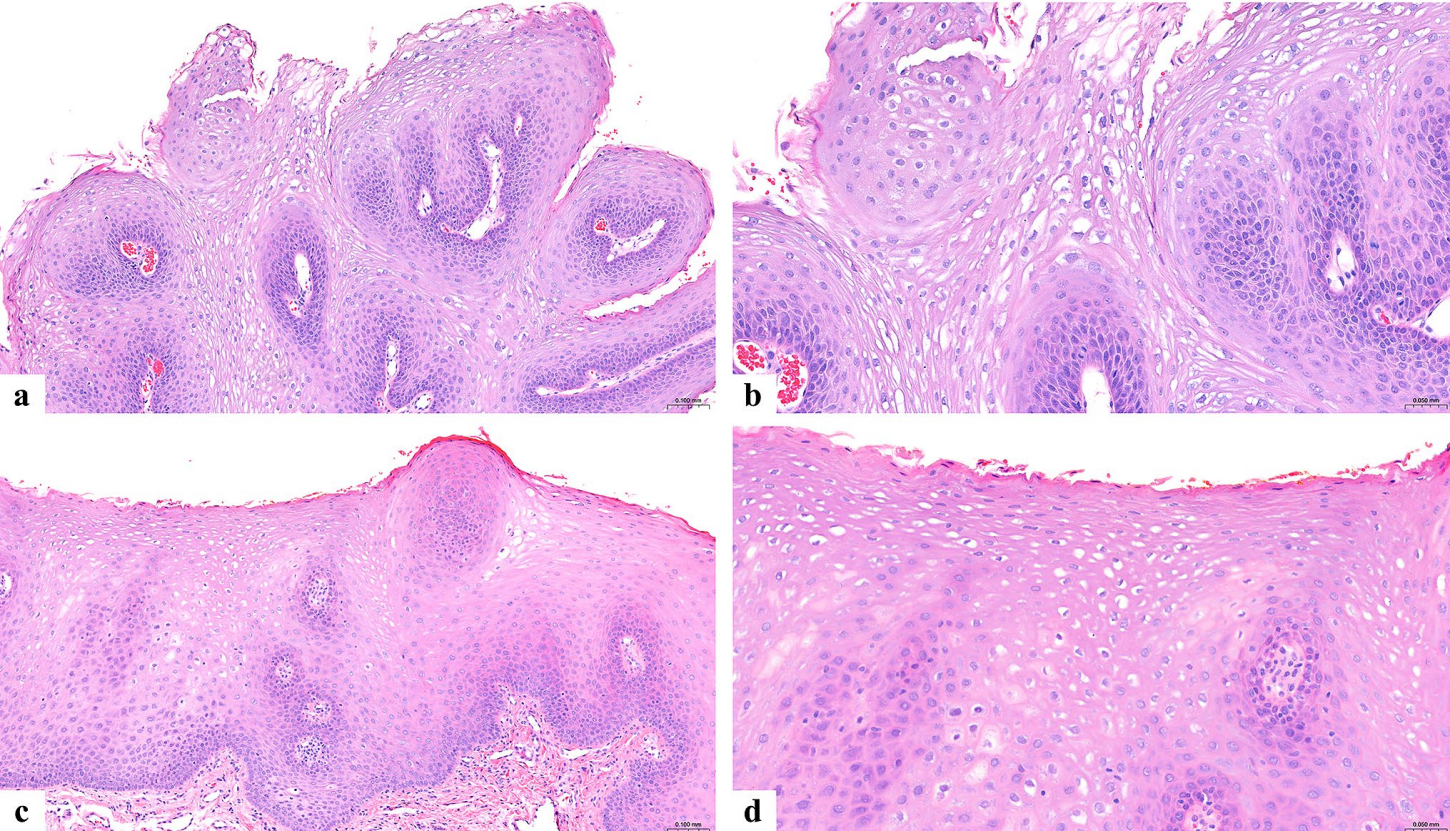
Histopathologic findings from biopsies six years prior. **(a, b)** Floor of the mouth: acanthotic, parakeratinized stratified squamous epithelium with papillary projections and koilocytes in the superficial spinous layer (H&E, ×100 and ×200). **(c, d)** Labial mucosa: parakeratinized stratified squamous epithelium with acanthosis and koilocytes in the superficial spinous layer (H&E, ×100 and ×200)

Of note, six years earlier the patient underwent biopsies for small, asymptomatic nodules on the lower lip and floor of mouth. Histopathologic findings of the specimen obtained from the floor of the mouth demonstrated acanthotic, parakeratinized stratified squamous epithelium with papillary projections (Fig. 3a) and koilocytes in the superficial spinous layer (Fig. 3b), and was diagnosed as squamous papilloma. The labial mucosa biopsy demonstrated parakeratinized stratified squamous epithelium with acanthosis (Fig. 3c) and koilocytes in the superficial spinous layer (Fig. 3d). At that time, the lesions were limited in extent, and no relevant medical history or medication use was documented on the biopsy submission form.

### Discussion and Literature Review

Fingolimod therapy alters immune cell trafficking by binding to S1P receptors, reducing circulating lymphocytes, and thereby increasing susceptibility to infection [1]. Its immunosuppressive mechanism is believed to underlie the reported associations with a variety of opportunistic infections and virus-mediated conditions [2].

Emerging evidence suggests a possible link between fingolimod therapy and HPV-related lesions, including cutaneous and genital warts, vulvar and cervical dysplasia, and HPV16-associated oropharyngeal carcinoma [3–7]. These associations suggest that fingolimod may impair immune control of latent or subclinical HPV infections, allowing for viral reactivation or persistent proliferation. In some previously reported cases, resolution of HPV-related lesions occurred following fingolimod discontinuation or substitution with alternative multiple sclerosis therapy [4, 6], further suggesting a causal relationship. However, many of these patients also received concurrent lesion-directed treatments, such as surgical excision or topical therapy, in addition to fingolimod withdrawal [4, 6]. Therefore, it remains challenging to determine if lesion resolution primarily resulted from immune reconstitution or local therapeutic interventions. Clinical outcomes should be interpreted within the context of both systemic immunomodulation and local lesion management. Furthermore, recurrence of cutaneous warts during fingolimod therapy has been documented in some cases, with subsequent resolution and absence of recurrence after cessation of the drug [5]. These patterns provide additional support for a potential association between fingolimod and HPV-related epithelial lesions. Table 1 summarizes reported cases of fingolimod-associated HPV-related lesions across various anatomical sites. None of the previously documented cases indicated the specific fingolimod used by reporting the trade name.

**Table 1:** Summary of reported cases of HPV-associated lesions in patients receiving fingolimod therapy

Authors, year (reference)	Age (year)	Sex	Fingolimod duration at onset (month)	Site	Diagnosis	HPV type(s)	Fingolimod modification	Clinical outcome ^a^
Benedetti et al., 2018 [3]	50	M	72	Palatine tonsil	Papillary squamous cell carcinoma	HPV 16	Discontinue	NA
Jaafar et al., 2019 [5]	32	F	48	Genitals	Condyloma accuminata	NA	Discontinue	Resolution in 2 months
	34	F	84	Genitals	Condyloma accuminata	HPV 6, 61, 52	Discontinue	Resolution in 1 month
	38	F	Few months	Nasal mucosa	Verruca vulgaris	NA	Switch DMT	Resolution with no recurrence
	26	M	NA	Feet	Verruca vulgaris	NA	No change	Improved but not complete resolved
	34	F	12	Feet	Verruca vulgaris	NA	Switch DMT	Resolution with no recurrence
Triplett et al., 2019 [6]	NA	NA	58	Anus	Squamous cell carcinoma	NA	Discontinue	Metastasis
	NA	NA	17	Ankle	Verruca vulgaris	NA	Dose reduction	Reduced in size
	NA	NA	20	Feet	Verruca vulgaris	NA	No change	Persistent
	NA	NA	49	Face	Verruca filiformis	NA	Dose reduction	Resolution in 6 months
	NA	NA	51	Finger	Verruca vulgaris	NA	Discontinue	Resolution in 4 months
Mamardashvili et al., 2025 [7]	36	F	4	Genitals	Condyloma accuminata	HPV positive, type not specified	Switch DMT	Resolution in 2 months
Mhanna et al., 2021 [4]	Mean ± SD: 34 ± 7 (range: 22–47)^b^	F: 11, M: 5^b^	Mean ± SD: 48 ± 32.4 (range: 2.4–144)^b^	Feet and hand	Verruca filiformis	NA	NA	NA
				Feet and hand	Verruca filiformis	NA	NA	NA
				Feet and hand	Condyloma accuminata	NA	Switch DMT	Clinical improvement
				Genitals	Condyloma accuminata	NA	Switch DMT	No clinical improvement
				Genitals	Condyloma accuminata	NA	NA	NA
				Genitals	Condyloma accuminata	NA	NA	NA
				Cervix	LSIL	NA	Switch DMT	Clinical improvement
				Cervix	LSIL	NA	NA	NA
				Cervix	LSIL	NA	NA	NA
				Cervix	LSIL	NA	Switch DMT	Clinical improvement
				Cervix	HSIL	NA	NA	NA
				Cervix	HSIL	HPV 16	NA	NA
				Cervix	HSIL	NA	NA	NA
				Genitals, hand and feet	Condyloma accuminata, verruca vulgaris	NA	Switch DMT	Clinical improvement
				Genitals	Condyloma accuminata, HSIL	HPV 16	Switch DMT	Clinical improvement
				Genitals, hand and feet	Condyloma accuminata, verruca vulgaris, LSIL	HPV 16	NA	NA

Abbreviations: M, male; F, female; LSIL, low-grade squamous intraepithelial lesion; HSIL, high-grade squamous intraepithelial lesion; NA, not applicable; DMT, disease-modifying therapy

^a^Clinical outcomes reflect the combined effects of lesion-specific treatment and/or fingolimod therapy modification. Attribution of outcomes solely to fingolimod adjustment should be interpreted with caution

^b^Only aggregated demographic data were available for these 16 cases. Individual case-level age, sex, and fingolimod duration were not reported.

In the present case, the histopathologic finding of koilocytosis supports a viral cytopathic effect consistent with HPV infection, which was further confirmed by PCR-based detection of low-risk HPV types 6/11. These genotypes are the predominant types implicated in benign oral epithelial proliferations, including squamous papilloma and condyloma acuminatum [8]. Histopathologic findings showing the exophytic papillary epithelial proliferation with HPV 6/11 detection in this case overlaps with features commonly seen in these two entities. However, the extensive, multifocal, and coalescing nature of the lesions observed clinically in this patient were not typical for either squamous papilloma or condyloma acuminatum, making these diagnoses less likely. Low-risk HPV types 6/11 are also commonly associated with genital warts [9], which have been reported in patients receiving fingolimod therapy [4, 5, 7]. These observations suggest that individuals treated with fingolimod may be predisposed to HPV 6/11-related lesions. Only two previously reported cases of genital warts included molecular HPV testing: one demonstrated HPV 6 and 61 (low-risk types) as well as HPV 52 (high-risk types) [5], and another reported HPV positivity without genotype specification [7]. Most of the other reported cases did not include HPV genotyping [4, 5]. Therefore, direct comparison with the present case is limited, and this case expands the literature by providing molecular confirmation of HPV type involvement in an oral mucosal presentation during fingolimod therapy.

p16 is widely recognized as a surrogate marker for transcriptionally active high-risk HPV, due to its upregulation by E7 oncoprotein-mediated inactivation of the retinoblastoma (Rb) tumor suppressor pathway [10]. As such, strong and diffuse p16 expression is generally an indicator of high-risk HPV involvement, particularly in oropharyngeal squamous cell carcinoma [10, 11]. In this case, both p16 immunostaining and in situ hybridization for high-risk HPV 16 were negative, consistent with the molecular findings that showed no detection of high-risk HPV genotypes.

It should be acknowledged that the commercial HPV DNA genotyping assay (NeoGenomics Laboratories, Fort Myers, FL) used in this case detects only a predefined panel of HPV types (reporting low-risk 6/11 as a combined target without differentiating between them) and does not encompass all known low-risk HPV genotypes. Broader molecular profiling may provide a more comprehensive understanding of HPV type diversity and pathogenic potential in such lesions.

Reports of HPV-related epithelial lesions at various anatomic sites particularly in anogenital areas in patients undergoing fingolimod therapy have raised the question if HPV vaccination should be considered before initiating this medication [12]. The vaccine shows maximal efficacy when administered before exposure to HPV, therefore, it is recommended for children and adolescents [13, 14]. However, multiple sclerosis is typically diagnosed in early adulthood or later [1, 15], which may reduce the preventive benefit of HPV vaccination when administered later in life. Therefore, clinicians should be aware of this rare adverse effect of fingolimod and emphasize clinical monitoring in patients receiving fingolimod therapy [12]. Further investigation is needed to determine if pre-treatment HPV vaccination could reduce the risk of HPV-related disease in patients initiating fingolimod.

Over the course of fingolimod therapy, this patient’s oral lesions evolved from small, isolated nodules to more widespread papillomatous proliferations involving multiple mucosal sites. This clinical progression highlights the immunosuppressive potential of fingolimod, which may have facilitated persistent or reactivated HPV infection. In addition, no prior or concurrent HPV-related epithelial lesions were documented outside the oral cavity, reinforcing the possibility of a localized mucosal response in the context of systemic immunomodulation.

To our knowledge, this represents the first reported case of oral mucosal HPV-associated epithelial proliferation in a patient receiving fingolimod therapy with molecular confirmation of HPV infection. The patient was lost to follow-up before any therapeutic modification could be implemented or lesion progression could be monitored, therefore, the potential impact of fingolimod discontinuation on lesion regression remains unknown. The temporal association with fingolimod use, along with the clinical presentation, characteristic histopathologic findings, and molecular results, strongly suggests a causal relationship. However, definitive causality cannot be established without longitudinal observation or documented lesion response following medication cessation. This case highlights the importance of thorough medical history taking and raises awareness of the potential for HPV-related oral mucosal changes in patients receiving fingolimod therapy.

## Data Availability

No datasets were generated or analysed during the current study.
